# Oxytocin in bovine saliva: validation of two assays and changes in parturition and at weaning

**DOI:** 10.1186/s12917-021-02838-5

**Published:** 2021-04-01

**Authors:** Marina López-Arjona, Eva Mainau, Elena Navarro, María Dolores Contreras-Aguilar, Damián Escribano, Sandra V. Mateo, Xavier Manteca, José Joaquín Cerón, Silvia Martínez-Subiela

**Affiliations:** 1 Interdisciplinary Laboratory of Clinical Analysis, Regional Campus of International Excellence ’Campus Mare Nostrum’, University of Murcia (Interlab-UMU), University of Murcia, Campus de Espinardo s/n, Espinardo, 30100 Murcia, Spain; 2 School of Veterinary Medicine, Universitat Autònoma de Barcelona, Bellaterra, 08193 Barcelona, Spain

**Keywords:** Calves, Cow, Oxytocin, Saliva

## Abstract

**Background:**

The possible use of oxytocin in saliva as an indicator of positive emotions in bovine species has been poorly investigated. In the present study, two new assays (one using a monoclonal antibody and the other using a polyclonal antibody) for the measurement of oxytocin in bovine saliva were developed and validated. Also, the changes in oxytocin in saliva were explored in two different situations. One was around parturition, and for this purpose, saliva samples from 13 cows were collected at three different times: 7 days before the parturition, the day of parturition and 7 days after the parturition. The second situation was weaning and grouping of calves, and for this purpose, saliva from 25 calves was collected at three different times: 1 day before weaning, 2 days after weaning or milk withdrawal and 4 days after grouping calves.

**Results:**

In cows, oxytocin concentrations showed an increase on the day of parturition with both assays, while in calves, oxytocin concentrations showed a decrease 4 days after the grouping.

**Conclusions:**

The assays validated in this report could be used for the measurement of oxytocin in bovine saliva and detect changes in this analyte that can occur in different physiological or productive situations such as parturition and weaning.

## Background

Oxytocin is a hormone released by neurons from the neurohypophysis, which is involved in milk ejection and uterine contractions [1]. In addition to these physiological functions, oxytocin has a role in maternal behavior [2] and it is involved in positive social interactions [3, 4] and positive emotions in humans [5] and animals [6, 7].

Saliva is gaining importance as a sample for oxytocin measurements because it has the advantage, compared with serum or cerebrospinal fluid, of being obtained through a non-invasive way and does not cause any stress to the animal [8]. Recently, two new accurate and precise assays have been developed for oxytocin measurement in the saliva of pigs. One uses a monoclonal antibody, and it can measure the oxytocin liberated from proteins after a reduction-alkylation (R/A) procedure, and the other assay uses a polyclonal antibody and it can measure oxytocin linked to proteins [9].

Some studies have evaluated oxytocin in cows in different physiological situations. Suckling increases oxytocin in serum in both dams and calves, being these increases related to positive animal welfare [6, 10]. However, in saliva, Geburt et al. [11] indicated that oxytocin is not a valid biomarker for maternal behavior in cattle and Lurzel et al. [12] found that salivary oxytocin concentrations in cattle do not change consistently after interaction of 10 min with a familiar or unfamiliar human.

Cows and calves face stressful situations during their production cycle. The occurrence of stress and reduced animal welfare can cause decreases in production, poor reproductive performance, or an increase in disease susceptibility [13, 14]. Parturition in cows is recognized as a painful and stressful process [15], resulting in hyperadrenocortical activity [16], which increases plasma glucocorticoid [17]. Regarding the separation of calves from their mothers, Pérez et al. [18] found that it had a stressful effect on both the mothers and the calves, showing some behaviors associated with stress, like vocalizations and locomotor activity, and also increase in cortisol concentration and loss of weight. Moreover, the calves also need to adapt to new social companions, and the regrouping and mixing of unfamiliar animals might lead to an increase in agonistic interactions [19].

To the author`s knowledge, in cows, there are no studies reported about different forms of oxytocin in saliva, and about changes in oxytocin concentrations after some stressful situations, such as parturition and the separation of calves from their mothers. The purposes of this study were: (a) to optimize and validate two assays, one using a monoclonal antibody and the other using a polyclonal antibody for oxytocin, which can measure different oxytocin components in the saliva of other species such as pigs, for oxytocin measurements in bovine saliva. (b) To evaluate if oxytocin measured by these two assays can change in two situations in which variations in oxytocin concentrations would be expected, one affecting cows such as parturition, and one affecting calves such as weaning and grouping.

## Results

### Optimization of monoclonal and polyclonal assay protocol for oxytocin measurement in bovine saliva

**Fig. 1:**
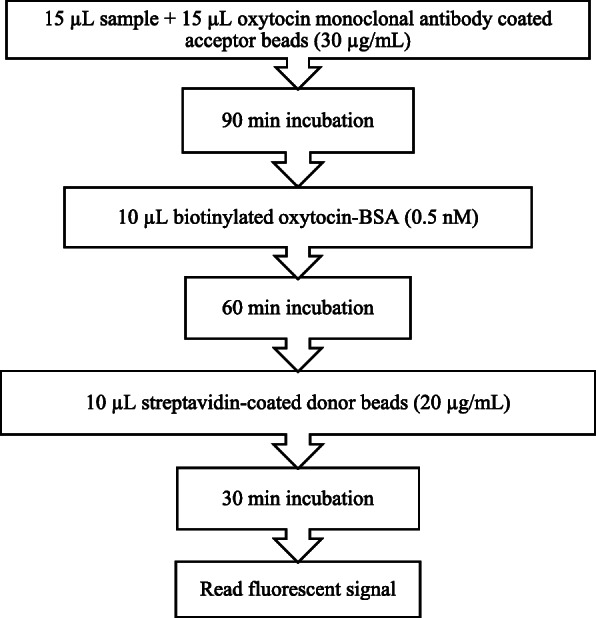
AlphaLISA monoclonal protocol for oxytocin measurement in bovine saliva

**Fig. 2:**
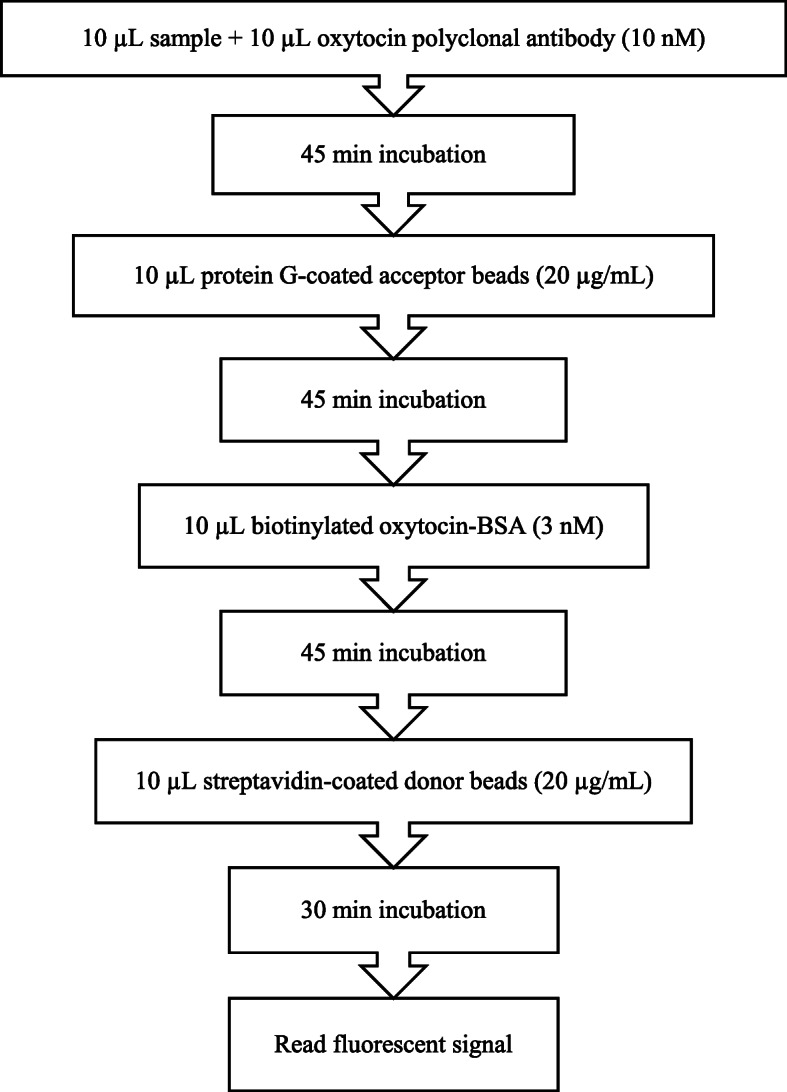
AlphaLISA polyclonal protocol for oxytocin measurement in bovine saliva

The protocols that resulted after optimization for monoclonal and polyclonal assays are shown in Figs. 1 and 2.

### Monoclonal and polyclonal assay validation

The monoclonal method showed intra-assay CVs of 2.3–8.1 % and inter-assay CVs of 10.7–13.4 %. Dilution of saliva samples resulted in linear regression equations with a correlation coefficient between 0.98 and 0.99. The results of the recovery obtained were between 93 and 120 %. The assay LD and LOQ were 7.1 and 56.8 pg/mL, respectively.

The polyclonal method showed intra-assay CVs of 5.1–15.1 % and inter-assay CVs of 9.3–16.2 %. Dilution of saliva samples resulted in linear regression equations with a correlation coefficient between 0.98 and 0.99. The results of the recovery obtained were between 99 and 120 %. The assay LD and LOQ were 4.7 and 2.4 ng/mL, respectively.

### Evaluation of the R/A effect

**Fig. 3:**
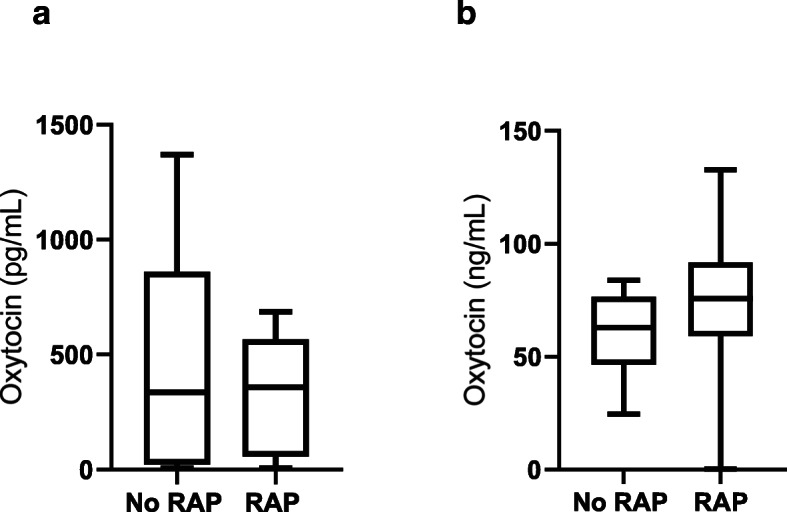
Results for oxytocin concentrations in saliva of cows before (No R/A) and after (R/A) the R/A treatment to the saliva samples with AlphaLISA monoclonal (**a**) and polyclonal (**b**) method. The plots show median (line within box), 25th and 75th percentiles (box) and minimum and maximum values (whiskers)

 Oxytocin concentrations with the monoclonal method showed no significant differences (*P* = 0.6237) between samples with the R/A procedure (358.6 pg/mL; 25-75th percentile: 56.8-567.4 pg/mL) and without the R/A procedure (337.0 pg/mL; 25-75th percentile: 22.6-860.1 pg/mL). In the case of the polyclonal method, oxytocin concentrations showed no differences (*P* = 0.0707) between samples with the R/A procedure (75.8 ng/mL; 25-75th percentile: 59.1–91.9 ng/mL) and without the R/A procedure (62.9 ng/mL; 25-75th percentile: 46.4–76.5 ng/mL). These results are shown in Fig. 3.

When buffers were analyzed as samples, they gave a result under the detection limit before and after the R/A procedure in the two assays.

The correlations between assays and with or without R/A procedure are shown in Table 1. The monoclonal method showed a significant positive correlation between values with and without R/A procedure (*r* = 0.873; *P*< 0.0001), while the polyclonal method showed a no significant correlation (*r* = 0.162; *P* = 0.495) with and without R/A procedure. The monoclonal method without R/A procedure showed a significant positive correlation with polyclonal method without R/A procedure (*r* = 0.565; *P* = 0.009) and a no significant correlation with polyclonal method with R/A procedure (*r* = 0.056; *P* = 0.821).

**Table 1:** Table 1 Spearman correlation coefficients between the two methods without (no R/A) and with R/A treatment (R/A). Asterisks indicate statistical significance (*P < 0.05; **P < 0.01; ****P < 0.0001)

	AlphaLISA monoclonal no R/A	AlphaLISA monoclonal R/A	AlphaLISA polyclonal no R/A	AlphaLISA polyclonal R/A
AlphaLISA monoclonal no R/A		0.873****	0.565**	0.056
AlphaLISA monoclonal R/A	0.873****		0.523*	0.112
AlphaLISA polyclonal no R/A	0.565**	0.523*		0.162
AlphaLISA polyclonal R/A	0.056	0.112	0.162	

### Oxytocin concentrations in saliva of cows around parturition

**Fig. 4:**
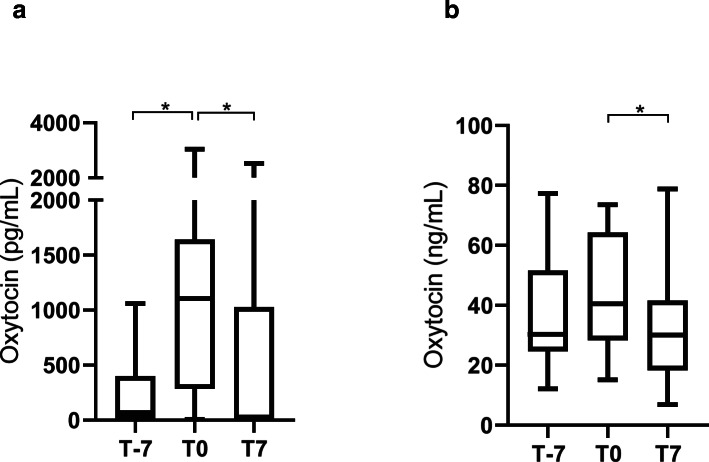
Results for oxytocin concentrations in saliva of cows at 7 days before the parturition (T-7), the day of parturition (T0) and 7 days after the parturition (T7) with AlphaLISA monoclonal (**a**) and polyclonal (**b**) methods. Asterisks indicate significant differences (*P < 0.05). The plots show median (line within box), 25th and 75th percentiles (box) and minimum and maximum values (whiskers)

When the monoclonal method was used in saliva samples, a significant increase was observed in T0 (1105.0 pg/mL; 25-75th percentile: 286.0-1643.0 pg/mL) compared with T-7 (69.1 pg/mL; 25-75th percentile: 7.13–401.5 pg/mL) and T7 (7.1 pg/mL; 25-75th percentile: 7.1–1028.0 pg/mL) (*P* = 0.0375 and *P* = 0.0306, respectively). When the polyclonal method was used in saliva samples, a significant increase was observed in T0 (40.5 ng/mL; 25-75th percentile: 28.4–64.3 ng/mL) compared with T7 (30.1 ng/mL; 25-75th percentile: 18.3–41.6 ng/mL) (*P* = 0.0189). T0 did not show significant changes when compared with T-7 (30.4 ng/mL; 25-75th percentile: 24.7–51.7 ng/mL) (*P* = 0.1821) (Fig. 4).

### Oxytocin concentrations in saliva of calves after weaning and grouping

When the monoclonal method was used, there were higher values for oxytocin before weaning (780.0 pg/mL; 25-75th percentile, 591.3-955.4 pg/mL) and after weaning time (795.0 pg/mL; 25-75th percentile, 657.3-952.3 pg/mL) than after grouping time (18.6 pg/mL; 25-75th percentile, 7.1-299.1 pg/mL) (*P* < 0.0001).

With the polyclonal method, there were higher values for oxytocin before weaning (107.2 ng/mL; 25-75th percentile, 81.9-130.7 ng/mL) and after weaning time (88.8 ng/mL; 25-75th percentile, 70.5-122.2 ng/mL) than after grouping time (64.6 ng/mL; 25-75th percentile, 37.9–85.5 ng/mL) (*P* = 0.0016 and *P* = 0.0068, respectively).

**Fig. 5:**
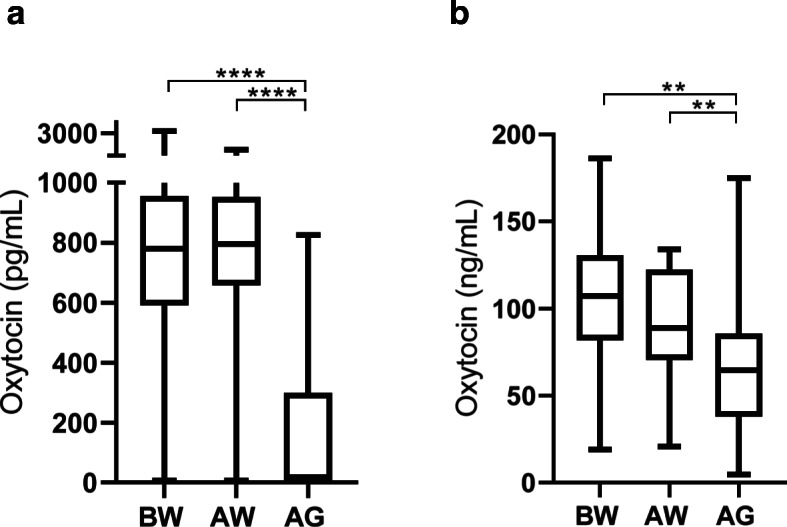
Oxytocin concentrations measured with monoclonal (**a**) and polyclonal (**b**) method in saliva of calves at 1 day before weaning (BW), 2 days after weaning or milk withdrawal (AW) and 4 days after grouping calves (AG) Asterisks indicate significant differences (**P < 0.01; ****P < 0.0001). Plots show median (line within box), 25th and 75th percentiles (box) and minimum and maximum values (whiskers)

Oxytocin concentrations at different times of sampling with the monoclonal and polyclonal methods are shown in Fig. 5.

## Discussion

Our findings indicate that oxytocin is present in bovine saliva as was reported in previous studies [11, 12] and that the assays used in our research can measure oxytocin in saliva samples with high precision and accuracy. These assays are based on the alphaLISA technology, which offers some advantages compared to ELISA (enzime-linked immunosorbent assay) kits, such as a lower sample volume and the no need for any washing step [20].

Regarding the effect of the R/A procedure with the monoclonal method, the results were similar to previous studies made with the same antibody in the saliva of pigs [9], with no significant changes and similar values of oxytocin concentrations before and after the R/A procedure. However, the results with the polyclonal method were not similar to previous studies in other species, since no significant changes were detected before and after the R/A procedure, while in pig saliva, the concentrations were significantly lower after the R/A procedure [9]. Therefore, according to these results, it could be postulated that the monoclonal method could evaluate the oxytocin that would be liberated after the R/A procedure without the need for this procedure in saliva samples [9]. However, more studies should be undertaken to determine which form of oxytocin is measured by the polyclonal assay, since contrarily to that reported in other species such as pig, in cows, this assay seems not to measure the oxytocin which is bound to proteins and it is not separated with the R/A procedure [9]. The high correlation found in alphaLISA monoclonal method between the values obtained before and after the R/A procedure leads us to use the samples without R/A treatment in the experiments made to evaluate the changes in this indicator in parturition and weaning.

The oxytocin concentrations detected by our polyclonal method were much higher than those for the monoclonal method. This could indicate that the polyclonal assay measures other different forms of oxytocin. Our monoclonal method yielded a similar range of values to a recent study in the saliva of cattle that used an ELISA kit, in which the saliva samples were extracted with solid-phase extraction [12]. Our However, our assay would have the advantage of not needing solid-phase extraction and would be faster and easier to perform.

In our experimental conditions, we found an increase in  salivary oxytocin at the day of parturition, being the magnitude of increase higher with the monoclonal method (16 fold) compared with the polyclonal method (1.3 fold). This increase could be because of oxytocin is involved in the mechanism of parturition [21, 22]. Although the parturition is a painful process for the cows [15] and would potentially decrease oxytocin levels, the high values obtained on the day of parturition would indicate that the role of oxytocin in the mechanisms of parturition predominates at this stage. In future studies, it would be interesting to evaluate oxytocin changes in dystocia or in other disorders related to parturition and explore if the concentrations would decrease as a consequence of the pain. The decrease in oxytocin found in our study in lactation would be in line with other studies that indicate that oxytocin secreted at nursing was lower that at parturition [23].

The decrease found in oxytocin concentrations in saliva in calves after grouping would indicate that the calves face a stressful situation at these times, with negative welfare implications [19, 24]. This data would suggest that oxytocin in saliva can reflect situations of negative emotions and be a marker of welfare in cows. This would be in contrast with other reports that found that oxytocin in saliva did not change in situations related to maternal behavior or interaction with humans [11, 12]. The different physiological situations evaluated and/or the assays used could be involved in these divergences.

There is some controversy about using oxytocin in serum as an indicator of stress or welfare due to its short half-life. However, in previous studies in humans, oxytocin persisted elevated during hours in saliva after its intranasal administration [25, 26], so oxytocin could have enough half-life in saliva to act as an indicator of stress or welfare.

## Conclusions

In conclusion, two new assays have been analytically validated for oxytocin measurement in bovine saliva. These assays could be used to measure oxytocin in bovine saliva and detect the changes that can occur in different physiological or productive situations such as parturition and weaning. In our particular conditions, the assay that uses a monoclonal antibody showed changes of higher magnitude and significance, and this would indicate that different results in oxytocin measurements in saliva of cows can be obtained depending of the assay used.

## Methods

### Optimization of monoclonal and polyclonal assay protocol for oxytocin measurement in cow saliva

The monoclonal antibody used for assay development was previously described in a report about oxytocin measurement in pigs [27]. The monoclonal assay, is a direct competition assay based on AlphaLisa (PerkinElmer, (MA, USA)) technology in which Acceptor beads coated to the monoclonal anti oxytocin antibody were used. In the assay optimization, different concentrations of biotinylated oxytocin (0–6 nM), acceptor beads (10–30 µg/mL) and streptavidin donor beads (with protein G) were tested (20–40 µg/mL) until an optimal concentration was found.

For the polyclonal assay, a polyclonal antibody previously described was used (López-Arjona et al., 2020). An indirect competition assay was developed, in which Acceptor beads are coated to protein G (PerkinElmer, Inc., USA). In the optimization of the assay, different concentrations of biotinylated oxytocin (0–6 nM) and polyclonal anti-oxytocin antibody (10–20 nM) were tested. Different concentrations of both Donor (PerkinElmer, Inc., USA) and Acceptor beads (with protein G) between 20 and 40 µg/mL were evaluated.

### Monoclonal and polyclonal assay validation

Oxytocin standards were prepared by diluting conjugated oxytocin to bovine serum albumin (oxytocin-BSA, Cusabio) in AlphaLISA Universal buffer. The standard curve was generated using eight standards at concentrations of 2400, 1200, 600, 300, 150, 75, 37.5 and 0 pg/mL in case of alphaLISA monoclonal method and 160, 80, 40, 20, 10, 5, 2.5 and 0 ng/mL in case of alphaLISA polyclonal method.

For analytical validation of the assays, the inter- and intra-assay coefficients of variations (CVs) were calculated for imprecision. Five replicates of each pooled sample with high, medium and low oxytocin concentrations were analyzed at the same time to determine the intra-assay precision. Five aliquots of each pool were stored in plastic vials at -80 ºC until analysis. These samples were thawed at room temperature and measured in duplicate five times over five different days for inter-assay precision.

The accuracy was evaluated by an assessment of linearity under dilution and recovery experiments. For the linearity evaluation, two samples were serially diluted from 1:2 to 1:128 with AlphaLISA Universal buffer. For the recovery test, different amounts (4000, 2000, 1000, 500 and 250 pg/mL in case of monoclonal method and 32, 16, 8, 4 and 2 ng/mL in case of polyclonal method) of commercially available conjugated oxytocin (oxytocin-BSA) were added to saliva samples with known oxytocin concentrations. The percentages of the measured to the expected oxytocin concentrations were calculated.

The limit of detection (LD) was obtained by the mean + two standard deviations (SD) of 12 replicated measurements of the AlphaLISA Universal buffer for monoclonal and polyclonal method. For the limit of quantification (LOQ), the lowest oxytocin concentrations that could be measured with < 20 % imprecision was calculated, so the saliva samples with a known oxytocin concentration (1300 pg/mL and 90 ng/mL for monoclonal and polyclonal method, respectively) were serially diluted from 1:2 to 1:128 with AlphaLISA universal buffer and analyzed five times at the same run for each dilution.

### Evaluation of R/A procedure

For evaluation of the R/A procedure in the saliva of cows, saliva samples were obtained from 20 cows (Holstein breed) from a farm of Murcia, Spain. The cows were all healthy and the saliva samples were obtained between 9.00 and 12.00 h. Each cow was sampled one time during the study. These saliva samples were measured by both assays with and without application of the R/A procedure.

The R/A procedure was carried out according to Brandtzaeg et al. [28] and 20 saliva samples from cows were used. At first, 100 µL of each sample was diluted into 200 µL 50 mM Tris-HCl (pH 8.0). Then 5 µL of 0.5 M DTT (dithiothreitol) (GE Healthcare Life Sciences, Marlborough, MA, USA) were added to 100 µL of the diluted saliva sample, mixed for 30 s and incubated at 37ºC for 45 min, followed by cooling to room temperature (22ºC). After that, 15 µL of 0.5 M IAM (iodoacetamide) (GE Healthcare Life Sciences, Marlborough, MA, USA) were added to each solution, mixed for 30 s and incubated at 22ºC in the dark for 20 min. Then, ice-cold 80 % ACN (acetonitrile) (Scharlab, S.L., Barcelona, Spain) in ultrapure water (Millipore) (v/v) was added to the solution, mixed for 30 s and centrifuged for 15 min at 14,000 rpm in a Sorvall ST 8R Centrifuge (Thermo Fisher Scientific, Waltham, MA, USA). After centrifugation, the supernatant was pipetted into a new Eppendorf and evaporated to dryness in a speed vac concentrator (Eppendorf Concentrator 5301) followed by reconstitution in 200 µL of PBS (phosphate-buffered saline). The treated samples were diluted 1:2 with buffer corresponding to each assay.

To evaluate if the R/A procedure could affect the assays used in our study independently from the effect that it has on bound oxytocin, the Universal assay buffer used for alphaLISA methods was processed using the R/A protocol and assayed as an unknown sample in each of the assays.

### Study of oxytocin changes around parturition

Saliva samples were obtained from 13 cows (between 3 and 8 years old, Holsteins) from a commercial farm. The cows were all healthy and with a body condition score between 2.5 and 4. The saliva samples were obtained between 9.00 and 12.00 h. Each cow was sampled three times during the study: 7 days before the parturition (T-7), the day of parturition (T0) and 7 days after the parturition (T7). The calves were separated from the cows the same day of parturition.

Saliva samples were collected using Salivette tubes (Sarstedt, Aktiengesellschaft & Co. Nümbrecht, Germany) containing a sponge (Esponja Marina, La Griega E. Koronis, Madrid, Spain) as previously described by López-Arjona et al. [29]. The cows were allowed to chew the sponge, which was clipped to a flexible thin metal rod for 1 min. Then, the sponges were placed into the Salivette tubes that were immediately centrifuged at 4500 rpm for 10 min at 4ºC. Saliva was then transferred into 1.5 mL eppendorf and stored at -80ºC until analysis.

The saliva samples of these experiments were analyzed by the monoclonal and polyclonal assays without R/A.

### Study of oxytocin changes at weaning and grouping

The samples used in this study were obtained from 25 calves (newborn female Friesian calves born from multiparous and primiparous dams) from a commercial farm. The study was performed from October 2018 to June 2019. The calves were sampled three times during the study: 1 day before weaning (BW), 2 days after weaning or milk withdrawal (AW) and 4 days after grouping calves (AG).

Saliva samples were obtained from all the calves included in the study. Saliva samples were collected from the calves using Salivette tubes (Sarstedt, Aktiengesellschaft & Co. Nümbrecht, Germany). Each tube contained a cotton swab, that was clipped through a Kocher clip, and calves were allowed to chew it during 1 min. Then, the cotton swab was placed in the tube and centrifuged at 6000 rpm for 12 min. Saliva samples (approximately 1–2mL per cotton swab) were stored in Eppendorf tubes and frozen at − 80 °C until analysis.

The saliva samples of these experiments were analyzed by the monoclonal and polyclonal assays without R/A.

The study was carried out in compliance with the ARRIVE guidelines [30].

### Statistical analysis

Medians and 25th-75th percentile were calculated by use of routine descriptive statistical procedures and computer software (Excel 2016, Microsoft Corporation, Redmond, Washington, USA). Statistical analyses were performed using Graph Pad Software Inc (GraphPad Prism, version 6 for Windows, Graph Pad Software Inc, San Diego, USA). The distribution of the studied variables was checked by using the Kolmogorov-Smirnov test and did not follow a normal distribution. Mann-Whitney test was used for evaluation of differences before and after the R/A procedure with each method, and the Spearman correlation coefficients were calculated to evaluate the correlation between the two methods before and after R/A procedure. A one-way Repeated Measures Analysis of Variance (ANOVA) followed by uncorrected Fisher’s Least Significant Difference (LSD) test was used, after log-transformation of data, to compare oxytocin values obtained in cows at different times (T-7, T0 and T7) and to determine if there were any significant differences in the values obtained in calves at different times (BW, AW and AG). Results were reported as median and 25th-75th percentile (in the text) and line-box plots (in Figures) and a *P* < 0.05 was considered significant.

## Data Availability

The datasets during and/or analyzed during the current study available from the corresponding author on reasonable request.
